# C3a Receptor Antagonist Ameliorates Inflammatory and Fibrotic Signals in Type 2 Diabetic Nephropathy by Suppressing the Activation of TGF-β/smad3 and IKBα Pathway

**DOI:** 10.1371/journal.pone.0113639

**Published:** 2014-11-25

**Authors:** Ling Li, Qinghua Yin, Xi Tang, Lin Bai, Jie Zhang, Shenju Gou, Hongping Zhu, Jingqiu Cheng, Ping Fu, Fang Liu

**Affiliations:** 1 Division of Nephrology, West China Hospital of Sichuan University, Chengdu, Sichuan, China; 2 Key Laboratory of Transplant Engineering and Immunology, Ministry of Health, Regenerative Medicine Research Center, West China Hospital of Sichuan University, Chengdu, Sichuan, China; The University of Manchester, United Kingdom

## Abstract

**Objective:**

Diabetic nephropathy (DN) is a serious complication for patients with diabetes mellitus (DM). Emerging evidence suggests that complement C3a is involved in the progression of DN. The aim of this study was to investigate the effect of C3a Receptor Agonist (C3aRA) on DN and its potential mechanism of action in rats with type 2 diabetes mellitus (T2DM).

**Methods:**

T2DM was induced in SD rats by a high fat diet (HFD) plus repeated low dose streptozocin (STZ) injections. T2DM rats were treated with vehicle or C3aRA for 8 weeks. Biochemical analysis, HE and PAS stains were performed to evaluate the renal function and pathological changes. Human renal glomerular endothelial cells (HRGECs) were cultured and treated with normal glucose (NG), high glucose (HG), HG+C3a, HG+C3a+C3aRA and HG+C3a+BAY-11-7082 (p-IKBα Inhibitor) or SIS3 (Smad3 Inhibitor), respectively. Real-time PCR, immunofluorescent staining and western blot were performed to detect the mRNA and protein levels, respectively.

**Results:**

T2DM rats showed worse renal morphology and impaired renal function compared with control rats, including elevated levels of serum creatinine (CREA), blood urea nitrogen (BUN) and urine albumin excretion (UACR), as well as increased levels of C3a, C3aR, IL-6, p-IKBα, collagen I, TGF-β and p-Smad3 in the kidney of T2DM rats and C3a-treated HRGECs. In contrast, C3aRA treatment improved renal function and morphology, reduced CREA, UACR and the intensity of PAS and collagen I staining in the kidney of T2DM rats, and decreased C3a, p-IKBα, IL-6, TGF-β, p-Smad3 and collagen I expressions in HRGECs and T2DM rats.

**Conclusion:**

C3a mediated pro-inflammatory and pro-fibrotic responses and aggravated renal injury in T2DM rats. C3aRA ameliorated T2DN by inhibiting IKBα phosphorylation and cytokine release, and also TGF-β/Smad3 signaling and ECM deposition. Therefore, complement C3a receptor is a potential therapeutic target for DN.

## Introduction

Diabetes mellitus (DM) is a major and increasing health problem worldwide [1]. Diabetic nephropathy (DN) is one of the most important causes leading to end-stage renal disease, which affects 15–25% of T1DM patients and 30–40% of T2DM patients [2],[3]. Multiple factors are involved in the pathogenesis of DN, including advanced glycation end products (ACEs), protein kinase C (PKC), transforming growth factor (TGF-β) and oxidative stress [4]–[6]. Recent studies have shown that T1DM patients with nephropathy had higher levels of mannose-binding lectin (MBL) [7], and T2DM patients with high level of MBL at baseline had a significantly increased risk of developing albuminuria [8], suggesting that the complement system is involved in the progression of DN.

The complement system serves as a part of the innate immune system [9],[10], with inappropriate activation of complement pathways leading to kidney damage [11]–[13]. The complement system mediates the progression of renal disease via both immune and non-immune pathways [10]. C3a is a small fragment derived from complement C3, which can bind to the G protein-coupled C3a receptor (C3aR) [14]. C3aR is expressed by various cells, including cells of hematopoietic origin such as neutrophils and monocytes, but also non-hematopoietic cells such as renal proximal tubular epithelial cells (PTECs) [14]. C3a was shown to induce anaphylatoxic reactions and recruitment of inflammatory cells [10]. Previous studies reported the increased expression of C3 in the glomeruli of diabetic mice and rats, and diabetic rats showed greater intensity of C3 staining in the renal mesangium when compared with controls [9]. We have previously shown that C3a is a pro-fibrotic factor, which can induce epithelial-myofibroblast transdifferentiation (EMT) in human renal proximal tubular epithelial (HK-2) cells via activation of the TGF-β1/CTGF pathway [15].

Glomerular endothelial cells (GECs) are characterized by fenestrations (60–80 nm transcellular holes) in the peripheral cytoplasm, which occupy a large proportion of the surface of glomerular filtration barrier (GFB), and play a key role in mediating the permeability of GFB to water and small molecules [16]. Loss or a reduced number of GECs will lead to dysfunction of glomerular filtration. Increasing evidence indicates that endothelial dysfunction is an early feature of DN [17]–[18]. It has been reported that GEC injury is already present in the normoalbuminuric stage of DN before podocyte injury [17]. It also contributes to the reduction of glomerular filtration rate (GFR) in DN [18]. Despite the role of complement-induced endothelial injury being proposed in other diseases, the specific effect of complement on GECs during the development of DN is incompletely known. Therefore, the effect of complement C3a on GECs was elucidated.

It is well documented that enhanced inflammatory responses occur in both animal models and human DN [19]. Nuclear factor kappa B (NF-κB) is a key transcription factor that controls the progression of inflammation. Many pro-inflammatory cytokines are transcriptionally regulated by NF-κB and are implicated in the pathogenesis of DN [19]–[20]. TGF-β/Smads are a key mediator of renal fibrosis and play a critical role in the progression of DN [21]. TGF-β/Smads mediate renal fibrosis by stimulating extracellular matrix (ECM) production and inducing the transformation of tubular epithelial cells (TECs) to myofibroblasts through EMT. However, the effect of C3aR blockade on the inflammatory and fibrotic pathways remains unclear.

To investigate the effect of complement C3a receptor blockade on T2DN, we treated rats with T2DM with C3aRA, and analyzed the morphological and functional renal changes. To further explore the underlying molecular mechanism, the expression of inflammatory and fibrotic signaling molecules was analyzed in human renal glomerular endothelial cells (HRGECs).

## Materials and Methods

### Ethics statement

Animal experiments were performed with the approval of the Animal Care and Use Ethics Committee of Sichuan University.

### Animal experiments

Male Sprague-Dawley (SD) rats (aged 6 weeks, 180–200 g) were purchased from the Laboratory Animal Centre of Sichuan University and kept under standard conditions at a temperature of 22±2°C, with a 12-h light/12-h dark cycle and relative humidity of 40–60%. T2DM was induced in rats using a high fat diet (HFD)+low-dose STZ method as previously reported [22]. All rats were allocated to one of two dietary regimens for an initial period of 6 weeks: normal pellet diet (NPD, n = 6) or high-fat and high-sugar diet (HFD), containing regular diet plus 27.3% lard, 54.6% sucrose, 16.4% cholesterol, and 1.6% sodium cholate [w/w], n = 18). Then, HFD rats with high HOMA-IR (fasting plasma glucose [mmol/L]×fasting insulin [mIU/L]÷22.5) were defined as insulin resistant, and were injected with repeated low-dose STZ (four doses of 25 mg/kg, Sigma, St Louis, MO, USA) in citrate buffer (pH = 4.5) after overnight fasting. NPD animals were injected with citrate buffer (1 ml/kg). The rats with fasting glucose levels ≥16.7 mmol/l at 72 h after STZ injection for three consecutive tests were used for the study. The rats with established T2DM were divided into two groups: T2DM+vehicle group (n = 6), T2DM+C3aRA group (n = 6). T2DM+vehicle and T2DM+C3aRA group was intraperitoneally injected daily with PBS or C3aRA (1 mg/kg, Merck, Darmstadt, Germany, dissolved in PBS) respectively for 8 weeks.

### Sample collection

Blood, urine and renal tissue samples were collected from rats after 8 weeks of treatment. Prior to sacrifice, rats were kept individually in metabolic cages and 24 h urine samples were collected. Rats were anesthetized with an intraperitoneal injection of sodium pentobarbital (40 mg/kg) and blood samples were collected by heart puncture. Their kidneys were removed and weighed, then the left kidney was fixed in 4% paraformaldehyde and the right kidney was snap frozen in liquid nitrogen then stored at −80°C for future use.

### Biochemical Measurements

Clinical biochemical analysis was performed on a biochemistry autoanalyzer (Cobas Integra 400 Plus, Roche, Basel, Switzerland) using commercial kits, and the following parameters were measured: fasting blood glucose (FBG), total cholesterol (TC), triglyceride (TG), blood urea nitrogen (BUN), serum creatinine (CRE), urinary protein excretion (ALB).

### Cell culture

Human renal glomerular endothelial cells (HRGECs) were purchased from SienCell Research Laboratories (San Diego, CA, USA). HRGECs were cultured in endothelial cell medium (ECM, ScienCell) supplemented with 5% fetal bovine serum (FBS, ScienCell), 1% endothelial cell growth supplement (ECGS, ScienCell) and 1% penicillin/streptomycin (ScienCell) at 37°C in a humidified incubator with 5% CO_2_. Cells were harvested with 0.25% trypsin (Gibco, Life Technologies, Carlsbad, CA, USA) at approximately 80% confluence, and the cells used within six passages experiments. Six groups were included in this study: normal glucose (NG, 5 mmol/L), high glucose (HG, 25 mmol/L), HG+C3a (50 nmol/l, Merk), HG+C3a+C3aRA (1 µmol/L, Merk), HG+C3a+SIS3 (Smad3 Inhibitor, 1 µmol/L, Santa Cruz, Dallas, TX, USA) or HG+C3a+BAY-11-7082 (BAY, p-IKBα Inhibitor, 5 µmol/L, Sigma). HRGECs were transferred to serum-free medium 24 h prior to treatment, and then treated with the above conditions for 3 or 5 days as indicated. Cells were then harvested for further analysis by RT-PCR and western blot.

### Real-time PCR

Total RNA was isolated from HRGECs using Trizol reagent (Takara, Shiga, Japan) according to the manufacturer's instructions. Total RNA was dissolved in RNase-free water and its concentration measured on a microspectrophotometer (Nanodrop 2000, Thermo Scientific Inc., MA, USA). The quality of RNA was determined by agar gel electrophoresis. cDNA was synthesized from RNA using a commercial kit (Bio-Rad, Hercules, CA, USA). Primer sequences are given in Table S1. PCR reactions were carried out in a volume of 20 µl on a CFX96 Real-Time PCR System (Bio-Rad) with SYBR Green kit (Tli RNaseH Plus, Takara), followed by melting curve analysis to distinguish the specific and non-specific PCR products. The relative expression of each gene was calculated using the delta-delta Ct method with GAPDH as a reference gene.

### Histological examination

Renal tissues were fixed in 4% paraformaldehyde, embedded in paraffin then 4 µm sections cut and stained with H&E, PAS and IHC staining for Collagen I (Calbiochem, Merck Millipore, MA, USA), C3a and C5b-9 (both from Abcam, MA, USA). For immunofluorescence (IF) staining, fresh frozen 4 µm sections of renal tissue were first fixed in 4% paraformaldehyde, permeabilized in 0.1% Triton X-100 for 30 min, and incubated with primary mouse anti-C3a (Abcam) and anti-IL-6 (Santa Cruz) antibodies diluted in 2% BSA in TBST at 37°C for 2 h, then overnight at 4°C overnight. Double-IF staining with rabbit anti-C3aR (GeneTex Inc., CA, USA), mouse anti-p-IKBα (Abcam, MA, USA), mouse anti-TGF-β (Calbiochem, Merck Millipore, MA, USA) and rabbit anti-p-Smad3 (Santa Cruz, CA, USA) with the endothelial cell markers mouse anti-CD31 (Abcam, MA, USA) or rabbit anti-CD31 (Bioworld Technology Inc., MN, USA) was also performed. The sections were washed with PBS and incubated with diluted fluoresce-conjugated secondary antibodies including goat anti-rabbit IgG/TRITC (Merck Millipore, Billerica, MA, USA) and goat anti-mouse IgG/FITC (Millipore) at 37°C in the dark for 1 h, and then stained with DAPI (Calbiochem). The micrograph of stained sections was acquired on a confocal microscope (Fluoview 1000, Olympus, Tokyo, Japan) with FV10-ASW software (version 1.7, Olympus), and morphologic analysis of images was done with Image J software. The double-blind experiment was carried out in the histological examination, and the person who evaluated the morphologic changes was blinded to the researcher who performed the treatment.

### Western blot

The cultured cells were lysed in RIPA buffer plus protease inhibitor (PMSF). Total cellular protein was collected by centrifugation, and the concentration determined by the BCA method. Proteins were first separated by SDS-PAGE and then transferred to PVDF membranes (0.45 µm, Millipore). The PVDF membranes were washed with TBST, blocked for 1 h with 5% skim milk powder dissolved in TBST, and incubated with primary antibodies against TGF-β (Calbiochem, MA, USA), Smad3 (Santa Cruz, CA, USA), p-Smad (Santa Cruz, CA, USA), Collagen I (Merck Millipore, MA, USA), C3a (Abcam, MA, USA), C3aR (GeneTex Inc., USA), IL-6 (LSBio, Seattle, WA, USA), p-IKBα (Abcam, MA, USA) and IKBα (Santa Cruz, CA, USA) at 4°C overnight with dilutions recommended by the manufacturer. β-actin was used as an internal reference. The PVDF membranes were washed with TBST and incubated with horseradish peroxidase (HRP) conjugated secondary antibodies at 37°C for 1 h. Protein bands were detected using chemiluminescence (ECL) reagent (Pierce, Thermo Scientific). The quantitative analysis of protein band density was performed on Quantity One (Bio-Rad).

### Statistical analysis

Statistical analysis was performed with SPSS software (version 11.5, IBM Corp., NY, USA), and descriptive statistics were presented as mean ± SD. Comparison between groups was analyzed with one-way analysis of variance (ANOVA) and Tukey's Post-hoc test, and p<0.05 was considered statistically significant.

## Results

### C3aRA ameliorates albuminuria and renal function in T2DM rats

The general and biochemical results of rats are shown in Table 1. T2DM rats had higher levels of BW, KW/BW, HOMA-IR, GLU, TC and TG than controls. However, there were no significant differences in these parameters between T2DM and C3aRA-treated rats. T2DM rats also showed impaired renal function when compared with the controls, including increased levels of BUN, CREA and UACR. In contrast, C3aRA treatment significantly reduced UACR and CREA levels in T2DM rats, and while BUN was slightly decreased in C3aRA-treated rats the difference was not significant.

**Table 1:** General and biochemical parameters in different groups.

Parameters	Control	T2DM	T2DM+C3aRA
Body weight (g)	306.8±21.0	377.3±42.3^a^	332.1±36.3^b^
KW/BW(g/kg)	3.45±0.35	4.44±0.28^a^	3.86±0.18^b^
HOMA-IR	0.78±0.26	2.55±0.75^a^	2.42±0.96^b^
GLU (mmol/L)	4.1±0.7	23.7±1.2^a^	24.7±1.85^b^
TC (mmol/L)	1.3±0.5	3.39±1.3^a^	3.41±1.5^b^
TG (mmol/L)	0.6±0.1	5.6±2.5^a^	5.4±1.6^b^
BUN (mmol/L)	5.6±0.5	16.4±4.2^a^	14.6±0.7^b^
CREA (µmol/L)	25.8±8.2	69.8±11.8^a^	54.7±5.3^b^ ^c^
UACR (mg/mmol)	0.6±0.2	8.45±3.8^a^	7.60±2.9^b^ ^c^

Note:

a control *vs* T2DM (p<0.05);

b control *vs* T2DM+C3aRA (p<0.05);

c T2DM *vs* T2DM+C3aRA (p<0.05).

### Effect of C3aRA on renal morphology in T2DM rats

**Figure 1:**
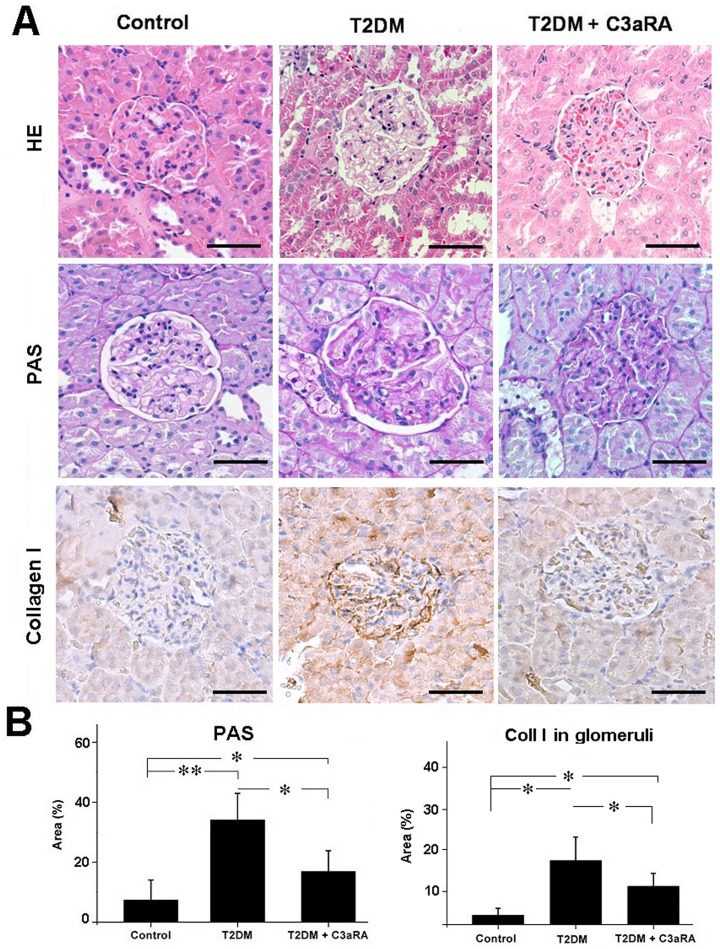
Histological analysis of glomerular injury of the different groups. (A) HE, PAS and IHC staining for collagen I (scale bar = 50 µm). (B) Quantitative analysis of PAS-positive area and collagen I level detected by IHC (*p<0.05, **p<0.01).

The histological results of kidneys from different treatment groups are shown Figure 1. T2DM rats showed clear renal lesions, including tubular hypertrophy, basement membrane thickening, mesangial proliferation and glomerulosclerosis. To assess the degree of glomerular sclerosis, PAS staining was performed. T2DM rats showed higher ratios of PAS-positive to PAS-negative areas than control rats. This ratio was significantly decreased in C3aRA-treated rats compared with T2DM rats. The collagen I level in glomeruli, which reflects the degree of matrix accumulation, was analyzed by IHC. Our results showed that C3aRA treatment markedly reduced glomerular collagen I disposition in T2DM rats.

### Effect of C3aRA on C3a/C3aR and collagen I levels

**Figure 2:**
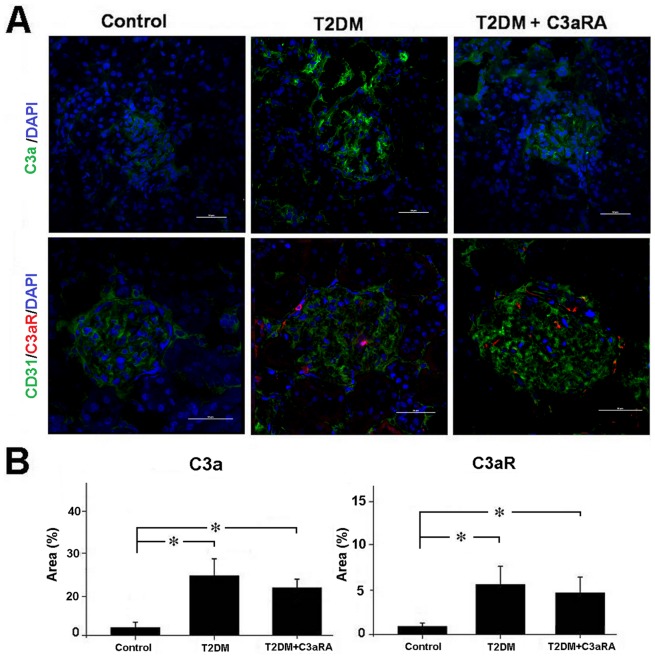
Immunofluorescent analysis of C3a and C3aR expression in kidney of different groups. (A) IF staining for C3a and double-IF staining of C3aR with CD31 (scale bar = 50 µm). (B) Quantitative analysis of C3a and C3aR level (*p<0.05, **p<0.01).

As shown in Figure 2, we observed positive staining of C3a and C3aR in glomeruli, and C3aR expression co-localized with CD31-positive GECs. T2DM rats showed higher intensity of C3a and C3aR staining than control rats. In contrast, C3aRA-treated rats showed decreased C3a levels when compared with T2DM rats, while C3aR level did not change significantly. These results indicated that C3aRA reduced renal C3a, but not C3aR, levels in T2DM rats. The level of C5b-9 was also analyzed (Figure S1). T2DM rats showed higher levels of C5b-9 than controls, while C3aRA had no effect on C5b-9 expression. These results indicate that the complement membrane attack complex (MAC) may be involved in the development of DN.

**Figure 3:**
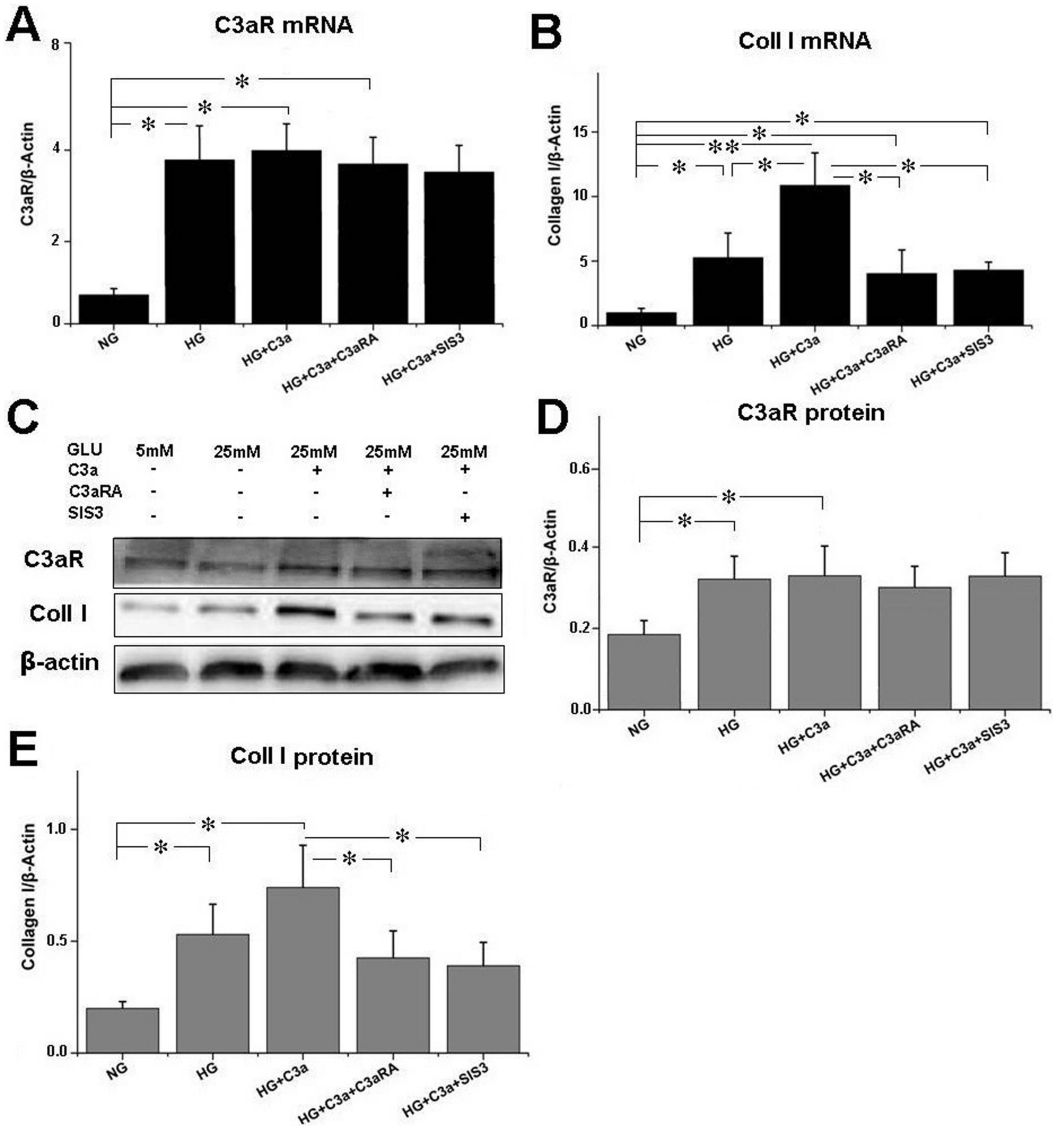
Real-time PCR for (A) C3aR and (B) collagen I expression in HRGECs. (C) Western blotting for C3aR and collagen I in HRGECs. Quantitative analysis of (D) C3aR and (E) collagen I level as detected by western blot (*p<0.05, **p<0.01).

The effect of C3aRA on HRGECs is shown in Figure 3. Compared with NG and HG group, HG+C3a treatment increased C3aR and collagen I level in HRGECs, while C3aRA and SIS3 (p-Smad3 inhibitor) reduced collagen I level in HG+C3a-treated HRGECs, although they did not affect C3aR level. These results indicated that C3aRA reduced collagen I expression, and that this effect was dependent on blocking C3a-C3aR interaction.

### Effect of C3aRA on IL-6 and p-IKBα levels

**Figure 4:**
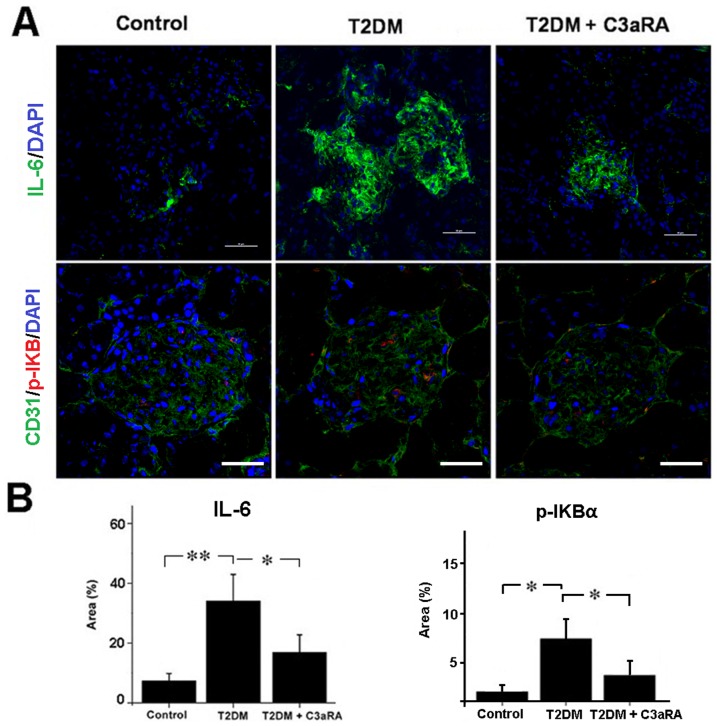
Immunofluorescent analysis of IL-6 and p-IKBα expression in the kidney of different groups. (A) IF staining for IL-6 and double-IF of p-IKBα with CD31 (scale bar = 50 µm), and (B) quantitative analysis of IL-6 and p-IKBα expression (*p<0.05, **p<0.01).

**Figure 5:**
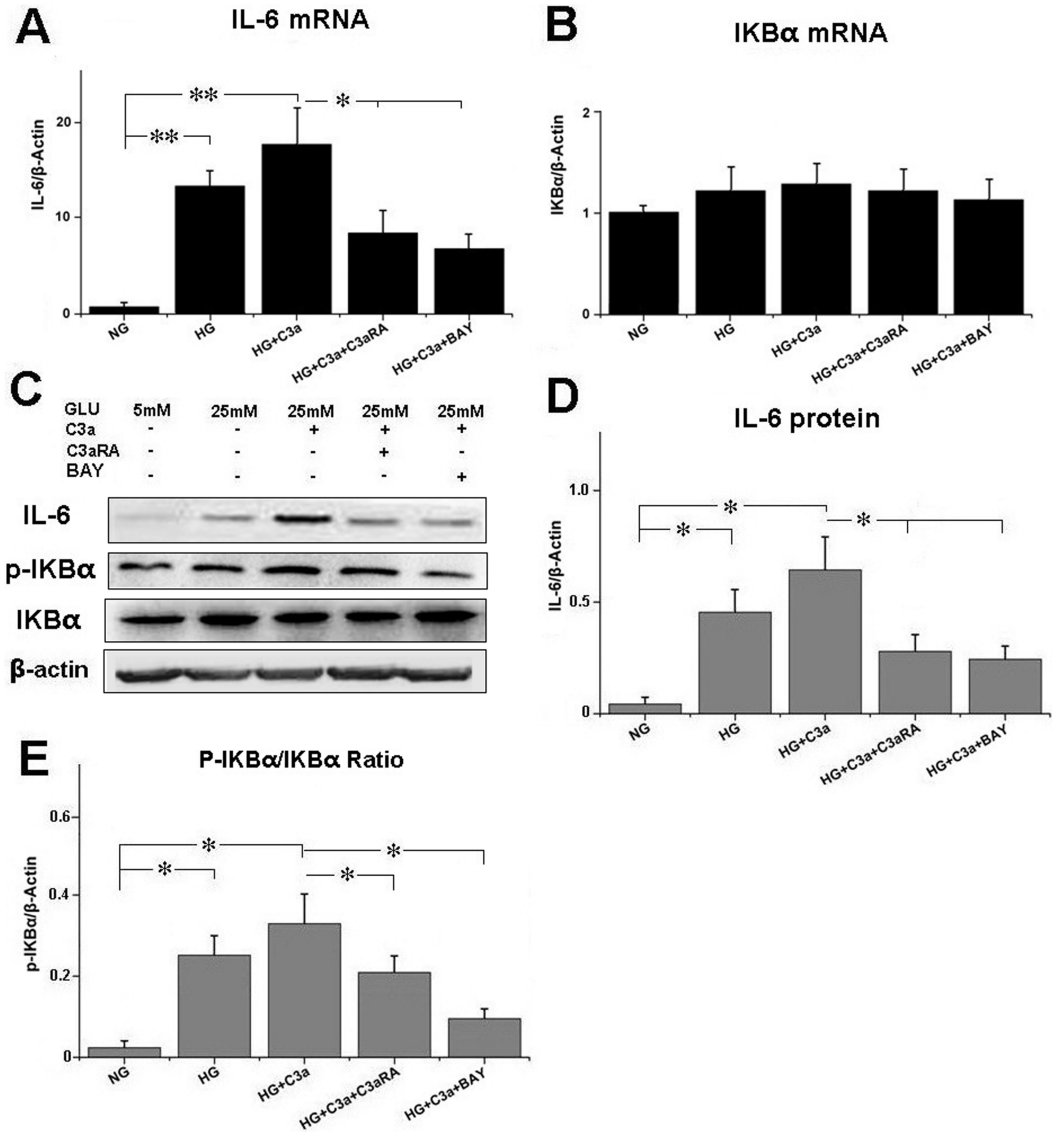
Real-time PCR for (A) IL-6 and (B) IKBα mRNA expression in HRGECs. (C) Western blotting for IL-6, IKBα and p-IKBα protein expression in HRGECs, and quantitative analysis of (D) IL-6 and (E) p-IKBα (*p<0.05, **p<0.01).

As shown in Figure 4, we observed positive immunostaining of p-IKBα in GECs of glomeruli. Compared with controls, T2DM rats showed higher levels of IL-6 and p-IKBα, while C3aRA treatment significantly decreased IL-6 and p-IKBα levels in T2DM rats. Furthermore, as shown in Figure 5, HG-treated HRGECs showed higher level of IL-6 than NG-treated HRGECs, and the addition of C3a further increased IL-6 expression. In contrast, C3aRA significantly reduced IL-6 level in HG+C3a-treated HRGECs, which was similar to the result obtained with BAY-11-7082 (a p-IKBα inhibitor). Despite the mRNA level of total IKBα was unchanged, the p-IKBα level was increased in HG+C3a-treated HRGECs. Both C3aRA and BAY reduced p-IKBα levels in HG+C3a-treated HRGECs, which suggested that C3aRA reduced IL-6 expression by inhibiting IKBα phosphorylation.

### Effect of C3aRA on TGF-β and p-Smad3 levels

**Figure 6:**
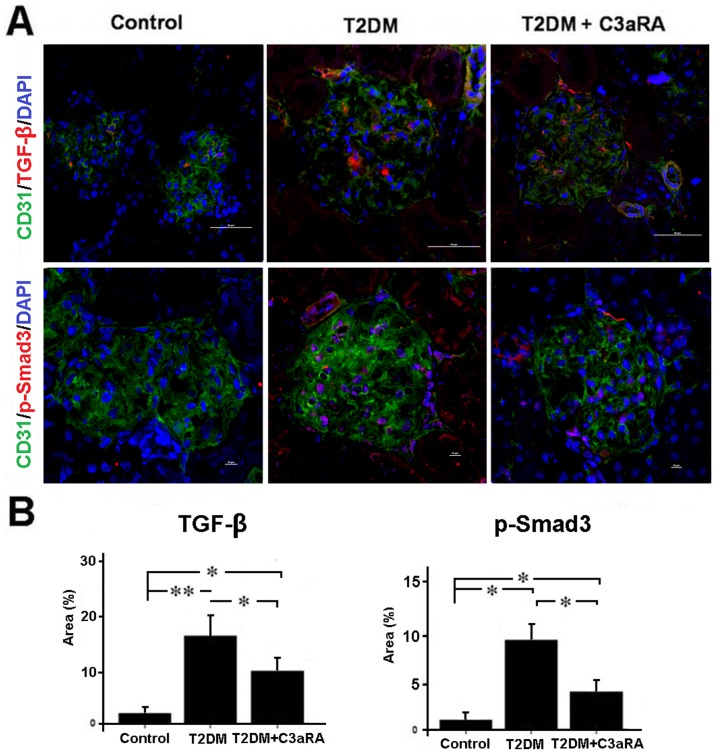
C3aRA inhibited TGF-β/Smad3 pathway in the kidney of T2DM rats. (A) Double-IF staining of TGF-β and p-Smad3 with CD31 (scale bar = 50 µm). (B) Quantitative analysis of TGF-β and p-Smad3 expression level (*p<0.05, **p<0.01).

**Figure 7:**
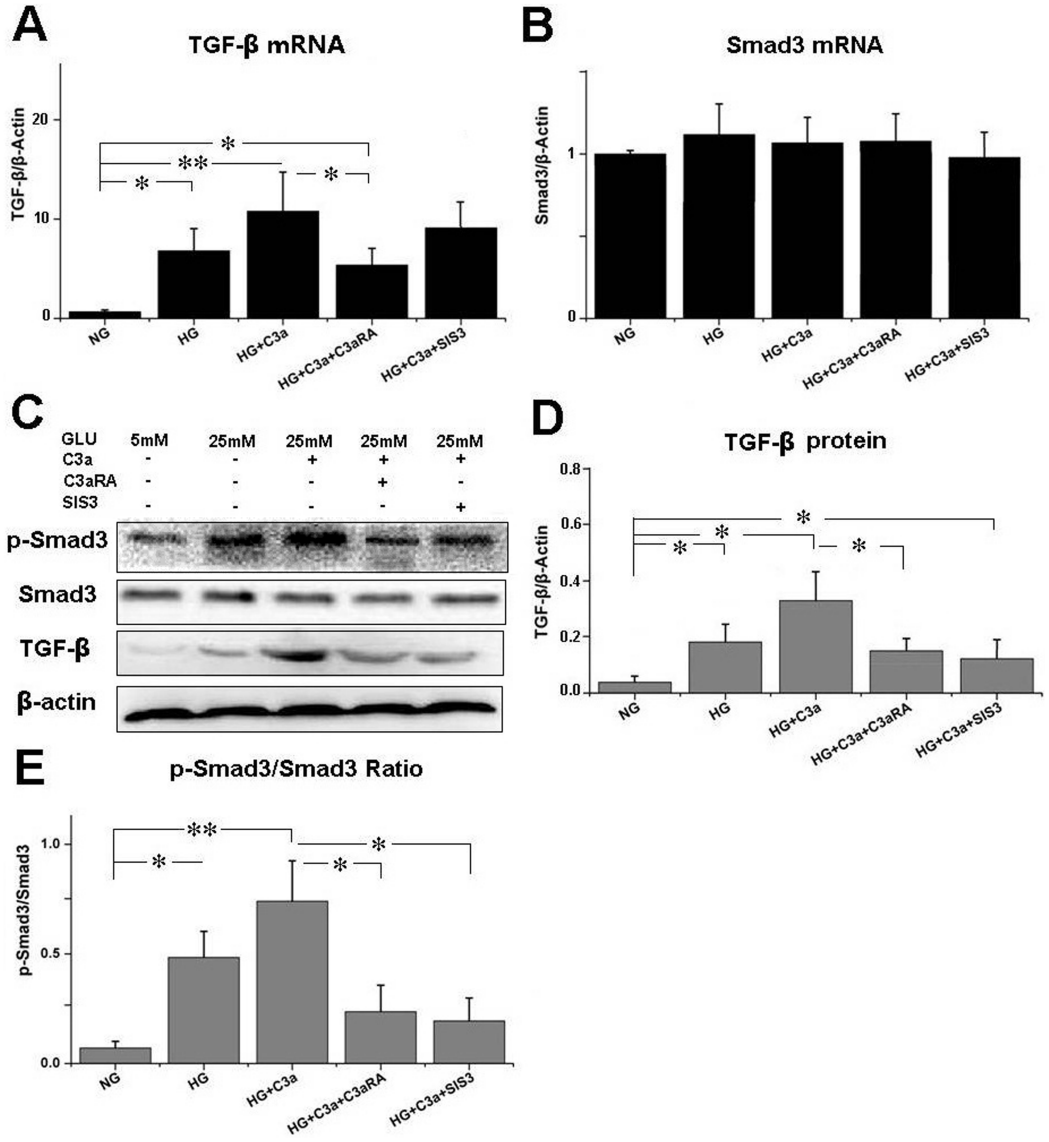
Real-time PCR for (A) TGF-β and (B) Smad3 mRNA in HRGECs. (C) Western blot for TGF-β, p-Smad3 and Smad3, and quantitative analysis of (D) TGF-β level and (E) p-Smad3/Smad3 ratio (*p<0.05, **p<0.01).

T2DM rats showed increased levels of TGF-β and p-Smad3 when compared with control rats, while C3aRA treatment reduced both TGF-β and p-Smad3 level in T2DM rats (Figure 6). These results indicated that C3aRA can inhibit the activation of the TGF-β/Smad3 pathway in T2DM rats. The effect of C3aRA on TGF-β/Smad3 in HRGECs was also analyzed (Figure 7). HG+C3a-treated HRGECs showed higher levels of TGF-β and p-Smad3 than the NG group. In contrast, C3aRA reduced TGF-β and p-Smad3 levels in HG+C3a-treated HRGECs, suggesting that the effect of C3aRA is partially mediated by inhibition of the TGF-β/Smad3 pathway.

## Discussion

Diabetic nephropathy is a serious complication of diabetes mellitus and has become the most common cause of end-stage renal disease worldwide. Although the relationship between complement C3a and renal disease has been reported in various studies [9],[12], the specific effect of complement in DN and the related molecular mechanism has not been well elucidated. In this study, we investigated the potential effect of C3a on glomeruli, especially on GECs in DN, and assessed whether blocking C3aR could ameliorate renal injury in a rat model of T2DM.

T2DM was induced in rats using a HFD+repeated low-dose injections of STZ. T2DM rats demonstrated hyperglycaemia, hyperlipidemia, increased BW, KW/BW and HOMA-IR, which are consistent with typical characteristics of T2DM in humans [1]. Our data showed that T2DM rats also had increased levels of BUN, CREA and UACR, which indicated renal dysfunction in T2DM rats. T2DM rats also showed obvious renal lesions, including increased intensity of PAS and collagen I staining in glomeruli, reflecting ECM deposition and renal fibrosis. These results demonstrated that the rat T2DN model was well established in our study.

A relationship between the complement system and renal disease has already been proposed in previous studies [23]. The complement-mediated renal injury can be induced via direct damage of membrane attack complex (MAC) to the cell surface, or through the activation of complement receptor and its downstream signals by complement fragments [10]. MAC such as C5b-9 can form transmembrane channels causing disruption of the target cell membrane and cell lysis, and C5b-9 has been involved in the glomerulonephritis such as membranous nephropathy [9]. C3a is a cleavage product of C3, which can induce inflammatory responses via binding to C3aR and activate its downstream signaling cascades [11]. In this study, T2DM rats showed increased level of C3a and C5b-9 in kidney, which was due to the abnormal activation of complement system in diabetes. C3aRA is a selective, high affinity and competitive antagonist of C3a receptor, which can inhibit C3a-induced internalization of C3aR [24]. T2DM rats treated with C3aRA showed clear improvement in renal function and pathology. C3aRA decreased the CREA and UACR in T2DM rats, while other biochemical parameters were not changed. These results suggested that C3aRA was able to ameliorate DN, and this effect was independent of mediating glucose and lipid metabolism. Although C3aRA slightly reduced renal C3a level in T2DM rats, the level of C5b-9 was not affected. This result indicated that C3aRA ameliorating renal injury mainly through inhibiting C3aR and its downstream signaling pathways, but not via directly reducing C5b-9 expression.

Previous studies indicate that the inflammatory response is a common pathogenic mechanism in chronic kidney disease (CKD), and that increased pro-inflammatory factors are associated with the progression of DN in human and animal models [25],[26]. Although the effect of C3a on the activation of neutrophils and its contribution to inflammatory responses is well documented, its specific effect on GECs in DN is not completely clear. The NF-κB pathway plays a crucial role in modulating pro-inflammatory factor expression, including cytokines, chemokines, growth factors and adhesion molecules, while inhibition of NF-κB signaling markedly reduces inflammatory responses [27],[28]. The phosphorylation and degradation of IKBα is a key step in the activation and translocation of the NF-κB complex to the nucleus [27]. We observed increased C3a and C3aR expression with increased p-IKBα and IL-6 in the kidney of T2DM rat, as well as increased p-IKBα and IL-6 in C3a-treated HRGECs, suggesting that C3a induces IL-6 release via p-IKBα and NF-κB activation. BAY 11-7082 is a selective inhibitor of IKBα phosphorylation, which specifically blocks the activation and translocation of NF-κB [29]. Both C3aRA and BAY could reduce p-IKBα levels in T2DM rats and C3a-treated HRGECs, which was associated with reduced IL-6. Therefore, our results demonstrated the anti-inflammatory effect of C3aRA in T2DM rats, and that this effect may be because of its inhibition of IKBα phosphorylation and NF-κB activation in GECs of the kidney.

Renal fibrosis is a hallmark of progressive renal disease leading to DN, which is characterized by excessive accumulation of ECM components, with collagen I being used as a common marker for ECM deposition [21],[30]. C3aRA treatment significantly reduced ECM deposition in T2DM rats, including decreased intensity of PAS and collagen I staining, which suggests C3aRA has a role in inhibiting renal fibrosis. To explore the potential mechanism, the C3a/C3aR and collagen I levels in HRGECs were also analyzed. We observed higher levels of C3a/C3aR and collagen I in the HG+C3a-treated group, while collagen I level was decreased in the C3aRA-treated group, suggesting that C3aR activation and its associated downstream signaling pathways contributed to the over-expression of ECM. A previous study reported the ability of C3a to induce phenotype change of PTECs by up-regulating TGF-β and collagen I [13], but its effects on GECs is still unknown. The TGF-β/Smad3 pathway plays a critical role in controlling ECM production and fibrosis [21]. After stimulation by TGF-β, Smad3 is phosphorylated at carboxyl terminal serine residues, then p-Smad3 forms a complex with Smad4 and translocates to the nucleus to induce pro-fibrotic gene expression [31]. In this study, we observed increased TGF-β and p-Smad3 in C3a-treated HRGECs and T2DM rats, suggesting that C3a plays an important role in the activation of TGF-β/Smad3 signaling and is a potential inducer of renal fibrosis. In contrast, C3aRA treatment inhibited TGF-β/p-Smad3 in T2DM rat and C3a-treated HRGECs, which showed similar effects to SIS3. SIS3 is an inhibitor of the phosphorylation of Smad3 [32], which also reduced p-Smad3 and collagen I in C3a-treated HRGECs. These results demonstrate that C3aRA can inhibit pro-fibrotic gene expression by inactivating TGF-β/Smad3 signaling in GECs. In addition, we also observed some positive staining of C3a/C3aR and the changes of its downstream signals in other glomerular and tubulointerstitium cells including podocytes and PTECs. As the previously studies had found that C3aR expressed on podocytes and PTECs [13],[33], our results also raised the possibility that C3aRA mediated the intracellular signals in these cells as well as in GECs. Therefore, further work will be needed to define the precise effects of C3aRA on the other type of renal cells such as podocytes and PTECs.

In conclusion, our study showed that C3a aggravates renal injury via up-regulation of the inflammatory and fibrotic responses in the T2DM rats. C3aRA treatment ameliorated renal injury, deceased albuminuria and ECM deposition in T2DM kidneys. C3aRA not only inhibited IKBα phosphorylation, but also inactivated TGF-β/Smad3 signaling in GECs. More importantly, our results demonstrated the anti-inflammatory and anti-fibrotic effect of C3aRA in the kidney of T2DN rats. Taken together, these findings suggest that C3aR activation potentially leads to the progression of DN, and thus C3aRA may serve as a novel therapeutic agent for DN.

## Supporting Information

Figure S1 **Immunohistochemistry staining for C3a and C5b-9 expression in kidney of different groups (scale bar = 50 µm).**
(TIF)

Table S1 **The sequences of primers for real-time PCR.**
(DOC)
