# Supplementary figures and images for: C3a Receptor Antagonist Ameliorates Inflammatory and Fibrotic Signals in Type 2 Diabetic Nephropathy by Suppressing the Activation of TGF-β/smad3 and IKBα Pathway
Source: PLoS One. 2014 Nov 25;9(11):e113639. doi: 10.1371/journal.pone.0113639 (PMC4244104; doi:10.1371/journal.pone.0113639)

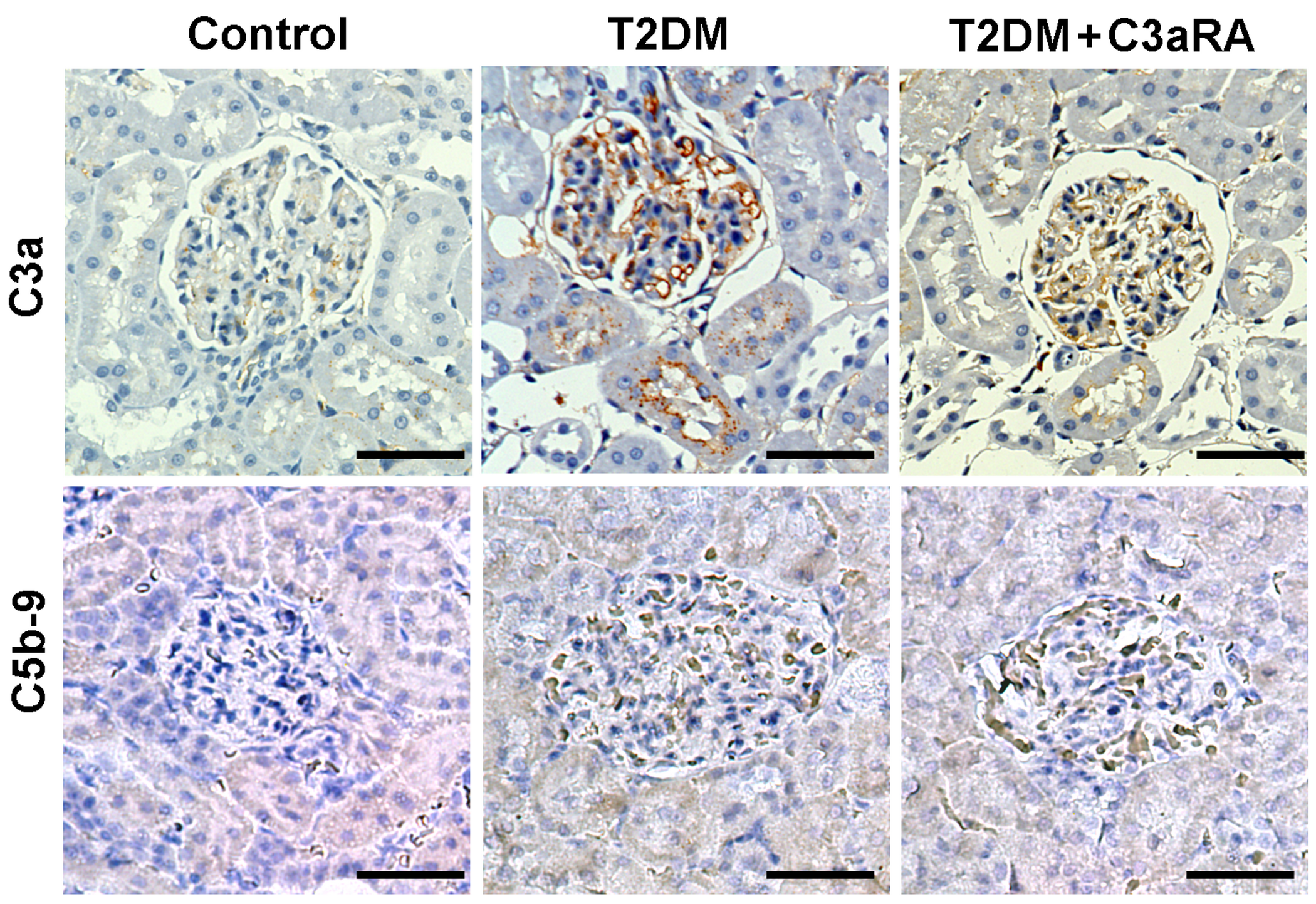

Supplement: Figure S1 — Immunohistochemistry staining for C3a and C5b-9 expression in kidney of different groups (scale bar = 50 µm). (TIF) [file pone.0113639.s001.tif]
