# Supplementary material for: C3a Receptor Antagonist Ameliorates Inflammatory and Fibrotic Signals in Type 2 Diabetic Nephropathy by Suppressing the Activation of TGF-β/smad3 and IKBα Pathway
Source: PLoS One. 2014 Nov 25;9(11):e113639. doi: 10.1371/journal.pone.0113639 (PMC4244104; doi:10.1371/journal.pone.0113639)
Supplement: Table S1 — The sequences of primers for real-time PCR. (DOC) [file pone.0113639.s002.doc]

**Table S1**

**Table S1 The sequences of primers for real-time PCR**

| Gene | Sequences |
| --- | --- |
| TGF-β | Forward 5-CGCATCCTAGACCCTTTCTC-3 |
|  | Reverse 5-TGTCTCAGTATCCCACGGAA-3 |
| Smad3 | Forward 5-CGCAGGTCCAAACCTAT-3 |
|  | Reverse 5-CGCTGGTTCAGCTCGTAGTA-3 |
| Col-1 | Forward 5-TGGTCCACTTGCTTGAAGAC-3 |
|  | Reverse 5-ACAGATTTGGGAAGGAGTGG-3 |
| IL-6 | Forward 5-AATGAGGAGACTTGCCTGGT-3 |
|  | Reverse 5-GCAGGAACTGGATCAGGACT -3 |
| IKBα | Forward 5-TCCGAGACTTTCGAGGAAAT-3 |
|  | Reverse 5-ACACGTGTGGCCATTGTAGT-3 |
| C3aR | Forward 5’-AGGCAATGGGCTGGTGCTGT- 3’ |
|  | Reverse 5’-CAGGAAGACACTGGCAAACAT-3’ |
| GAPDH | Forward 5’-CCTCAAGATCATCAGCAAT-3’ |
|  | Reverse 5’-CCATCCACAGTCTTCTGGGT-3’ |
